# Response: Commentary: Remote Electronic Training Aids; Efficacy at Deterring Predatory Behavior in Dogs and Implications for Training and Policy

**DOI:** 10.3389/fvets.2021.675005

**Published:** 2021-04-26

**Authors:** Jonathan J. Cooper, Daniel S. Mills, Lucy China

**Affiliations:** Animal Behaviour, Cognition and Welfare Research Group, School of Life Sciences, University of Lincoln, Lincoln, United Kingdom

**Keywords:** electronic training aids, dog training, animal welfare policy, efficacy of training, livestock worrying

In their commentary, (1) object to our conclusion that the use of e-collars are unnecessary in dog training (2). Their criticisms make 4 broad claims: firstly that the training approaches were not the most effective means of training with e-collars; secondly that the paper focused on measures of efficacy and did not present data on welfare; thirdly that the study did not include long term measures of efficacy; and fourthly our statistical approaches were not appropriate. Sargisson and McLean (1) also question whether the research should be used to inform policy decisions with regard to use of e-collars in dog training, although we were cautious not to make any specific recommendations regarding legislation in our paper. We shall deal with each of these objections in turn, placing the first three in the context of the research project as well as related published work, clarifying the statistical approaches as there appear to be misunderstandings by Sargisson and McLean (1) and finally relating the research to policy implications.

In China et al. (2), the e-collar trained group were exposed to a training approach whereby trainers first assessed the sensitivity of the dogs to the intensity of electrical signals and then seek to pair the application of the electric stimulus with pre-warning cues, such as hand signals, vocal signals, and lead pressure as well as collar born sonic and haptic (vibration) cues which can be delivered remotely by the trainer prior to delivery of an electrical stimulus. These approaches were advocated for improving the dogs' recall in the face of live distractors including sheep, poultry and other dogs as dogs had been referred for poor recall and associated problems, such as livestock worrying. The use of this approach to improve recall was compared with dogs referred for the same severity of recall related problems in two control groups, one of which was with the same trainers, but did not include use of e-collar stimuli and a second primarily reward-based training group who used food rewards and vocal cues to improve recall. The trainers using e-collars had been nominated by the trade organization representing e-collar manufacturers in UK (ECMA—http://ecma.eu.com/), and used the approaches developed and recommended by ECMA to improve recall in dogs (3), whereas the reward based trainers were members of the APDT and used approaches consistent with the training philosophy of that body (https://apdt.co.uk/code-of-practice-apdt/). Our main findings were that the reward-based training was more effective at improving recall during the training sessions with fewer training commands required, fewer errors during training and a shorter latency to respond to recall signals and sit commands.

Sargisson and McLean (1) state that this is not the most effective means of deterring dogs from predatory behavior. They advocate that the devices should be used at high intensities in a form of positive punishment to be most effective, whereas the ECMA approach we investigated could perhaps better fit the description of negative reinforcement as the use of pre-warning behaviors could allow dogs to modify their behavior to avoid exposure to the electric stimulus once they had been paired during early training. Sargisson and McLean (1) cite a small number of laboratory studies to support their claim as well as research in kiwi predation in New Zealand published by Dale et al. (4, 5). High intensity electrical stimuli without pre-warning cues have been investigated in a number of other studies of anti-predation training including studies with dogs and live sheep (6, 7). These support Sargisson and McLean's claims with regard to deterrence, however, it should be noted that these studies impose the training under controlled conditions, and the authors are generally cautious about translating their findings into field conditions. Dale et al. (4, 5) who investigated avoidance of a stuffed kiwi model, whilst supporting the potential to reduce kiwi predation in off lead dogs, *do not present any data on its effectiveness in the field*, whereas Christiansen et al. (6, 7) despite finding long term efficacy in deterring approach to sheep in dogs using e-collars, do not recommend the use of e-collars in dog training due to the challenges of consistently pairing the aversive with the target stimulus/behavior. This theme is taken up in Masson et al.'s (8) review, who recognized the potential efficacy of high intensity electric signals, but countered with concerns regarding dog's long and short term welfare and the dangers of unintended associations due to poor timing by operators [see also (9–11)]. An important study in this respect is the work of Schalke et al. (12) who investigated the impact of inconsistent application of training approach in controlled conditions, whereby beagles were exposed to the electric stimulus on approaching a stuffed toy. Whilst good timing was most effective and caused small rises in salivary corticosteroids, poor or random timing leads to poor training outcomes and elevated corticosteroids. The difficulty in ensuring consistent timing will be more challenging under field conditions with owners whose experience and competence in dog training will be variable, than conditions encountered under controlled training situations, and hence the risks of poor dog welfare and ineffective training elevated particularly where more intense signals are imposed (6, 8, 13).

Sargisson and McLean's point that China et al. (2) does not report long term measures of efficacy is correct as this report focuses on the efficiency of the training process. However, data on long term efficacy have already been published (14–16). These reported high levels of satisfaction by owners for all three training approaches and no difference in retention of learnt responses between the three groups. There was some evidence in feedback that owners of dogs who had received e-collar training were less confident of using the approaches and less satisfied with outcomes than those owners whose dogs had received reward-based training. These findings were broadly consistent with other reports of owner experience including that of Blackwell et al. (10) and were one of the reasons for re-examination of video records of dogs in training to gain a finer grained account of the methods used in training as well as the immediate consequences of the three training approaches. The conclusions of China et al. (2), should therefore be digested, with reference to previously reported findings regarding welfare consequences and long term efficacy in comparison to alternative approaches to training that address similar referred behaviors but without use of e-collars.

It is commonly stated by advocates of e-collar training, that their use is a last resort to deter highly motivated activities, such as predatory behavior (17). Whilst several studies have investigated efficacy under controlled situations, they rarely present evidence of translation to the field and do not compare use of e-collars with other training methods. For example, Dale et al. (4, 5) whilst they found efficacy in reducing interest by dogs in stuffed kiwis, when e-collars are used as a positive punishment, do not present any evidence that this translates to real live kiwi in the field and advocate further study (if ethically possible) under field conditions. As this form of training is required by New Zealand Department of Conservation before hunters can take their dogs off lead in kiwi populated areas (for example in order to control wild pig populations) it is to be hoped that this approach is effective at reducing kiwi predation in the field. There is, however, potential that the dogs make an association just to cues associated with the kiwi model or to other aspects of the controlled training situation, and as a consequence the modification of behavior does not generalize to live kiwis in field conditions. In addition to the lack of evidence of efficacy in real world conditions, the lack of investigation of other alternatives and the absence of real world testing does not rule out the possibility that more humane training can be just as effective. In contrast, the work reported in China et al. (2) shows improved levels of efficacy provided by reward-based training in a realistic training context, even by experienced trainers applying industry approved methods We believe this to be one of the most important aspects of this work, as it addresses a previous gap in the literature on comparative efficacy of different training methods, where it has been claimed the use of electronic collars is necessary (17).

Sargisson and McLean's point that China et al. (2) did not present data on dog welfare is correct, but again this does not recognize the already published work from our research group and others, which we cite in the paper [e.g., (10, 16, 18)], that address this issue. These report evidence of adverse behavioral responses during training, such as vocalizations and sudden body movements consistent with pain, as well as longer term changes in behavior that were consistent with anxiety or distress when returned to the training environment. The aim of China et al. (2) was a more focused assessment of modification of referred behavior during training than had been published previously. In addition to the measures on training efficacy, such as latency and accuracy of responses, we also collected data on the dogs' welfare related behavioral responses, which have now been published as part of China's thesis (19). Whilst not reported in China et al. (2), these measures provide further evidence of aversion during e-collar training including higher levels of lip-licking, yawning, paw-lifting and flight behaviors compared to the reward based training group. Furthermore, the vocalizations recorded in Cooper et al. (16) suggest that trainers did use intensities likely to have caused pain during training [see (10, 20)], so whilst ECMA endorsed training did not expose dogs to the highest settings available, it is likely to be less benign than Sargisson and McLean (1) suggest. These findings compliment the findings reported in China et al. (2), as well as our previously published research (14–16) which focused on dog welfare during and after training, which were cited in our study as evidence of welfare concerns. It is easy to cherry-pick specific results to make a case in support of a particular line of argument, but our aim has been to provide the most parsimonious explanation of all the data available. Thus, whilst individual behaviors might have alternative explanations, the totality of behaviors recorded are best explained as signs of reduced welfare during this training.

Sargisson and McLean (1) also draw attention to some concerns regarding analysis of data in our study. With regard to the ANOVA model, they suggest we should have used a repeated measure design. They are incorrect about this point, as we did use a repeated measure design, as specified by use of fixed and random factors. They express concerns we did not include interactions in our models; these had been included in initial analysis, however as none were found to be significant and their inclusion did not explain any variation in sample, we focused on main effects for simplicity. Leaving interactions in place would unnecessarily inflate the degrees of freedom in the analysis and increase the chance of spurious effects. Something they appear to be concerned about as they highlight a potential need to use a Bonferroni correction (or other correction approaches, such as sequential Bonferroni or false discovery rate adjustments) to accommodate multiple testing. However, we share the concerns of many researchers and statisticians concerning the blind use of dependence on arbitrary thresholds (21) and inappropriate use of such corrections (22). At the very least, their use needs to be carefully considered in relation to the issues arising from consideration of the impact of both Type I and Type II statistical errors. Our key findings were highly significant and would have still held even if Bonferroni had been inappropriately applied to the entire data set.

Finally, the question of whether the study should be used in advising on policy is worth addressing. Whilst one should be cautious about drawing conclusions that may influence policy makers on the basis of a single paper, the work adds detail to an already considerable body of evidence challenging the widespread use of electronic training aids in dog training, in particular that one of the potential contributing factors to poor welfare and lack of additional efficacy reported in other work is the difficulty of ensuring good timing of the stimuli under field conditions (8). We would therefore stand by our conclusion that conventional use of e-collars is unnecessary, and that this is a fair conclusion of our work as a whole, where policy makers seek to make evidence based policy decisions. It has been on that basis that The Welsh Assembly has upheld its 2010 ban on use of e-collars (23), whilst restrictions on use are being introduced across the UK and Europe (24, 25). Current legislation in New Zealand is consistent with our conclusions, as general use of e-collars by dog owning public is severely restricted (26) whilst allowing their use for specific proscribed purposes of high national priority, such as kiwi protection, in the absence of specific research to assess the efficacy of this form of training in the field compared with other forms of training and/or owner responsibility.

In conclusion, we endorse the resolution of the debate by policy makers through honest assessment of the available evidence rather than through the pressure applied by lobbyists. To this end, there is clearly a need for those arguing for the continued use of electronic collars to produce good quality research from the field, which addresses both the necessity and welfare impact of these devices, mindful of the ethical issues associated with this method of training. Especially given that the continued use of these devices is at odds with at least two (non-maleficence and autonomy) and potentially all four (beneficence and fairness) of the ethical principles widely accepted by those with a professional responsibility toward animals under their care

## Author Contributions

JC led the original research, co-authored the original paper, and was the lead author on response to commentary. LC conducted the re-analysis of video records as part of her master thesis and was first author on original paper. DM was the co-researcher in original project and co-author of original paper. All authors contributed to the article and approved the submitted version.

## Conflict of Interest

The authors declare that the research was conducted in the absence of any commercial or financial relationships that could be construed as a potential conflict of interest.
